# Crosstalk between circadian rhythms and the microbiota

**DOI:** 10.1111/imm.13278

**Published:** 2020-10-23

**Authors:** James Alexander Pearson, Florence Susan Wong, Li Wen

**Affiliations:** ^1^ Division of Infection and Immunity School of Medicine Cardiff University Cardiff UK; ^2^ Endocrinology, Internal Medicine School of Medicine Yale University New Haven CT USA

**Keywords:** circadian rhythms, immune system, Microbiota, pattern recognition receptors

## Abstract

Circadian rhythms influence daily molecular oscillations in gene/protein expression and aspects of biology and physiology, including behaviour, body temperature and sleep–wake cycles. These circadian rhythms have been associated with a number of metabolic, immune and microbial changes that correlate with health and susceptibility to disease, including infection. While light is the main inducer of circadian rhythms, other factors, including the microbiota, can have important effects on peripheral rhythms. The microbiota have been of significant interest to many investigators over the past decade, with the development of molecular techniques to identify large numbers of species and their function. These studies have shown microbial associations with disease susceptibility, and some of these have demonstrated that alterations in microbiota cause disease. Microbial circadian oscillations impact host metabolism and immunity directly and indirectly. Interestingly, microbial oscillations also regulate host circadian rhythms, and the host circadian rhythms in turn modulate microbial composition. Thus, it is of considerable interest and importance to understand the crosstalk between circadian rhythms and microbiota and especially the microbial influences on the host. In this review, we aim to discuss the role of circadian microbial oscillations and how they influence host immunity. In addition, we discuss how host circadian rhythms can also modulate microbial rhythms. We also discuss potential connections between microbes and circadian rhythms and how these may be used therapeutically to maximize clinical success.

## Graphical abstract

Microbial oscillations provide an important feedback system to both central and peripheral circadian clocks through the oscillations in microbial metabolites and ligands, which help to fine‐tune the oscillations and ensure the host can respond more quickly to environmental changes. The oscillations in microbiota are also important for modulating their own functions including chemotaxis, motility, adherence, DNA repair and metabolism. These microbial oscillations are vital for the finetuning of immune responses, which our review discusses in detail.

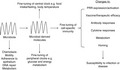

**Abbreviations**
BMAL1: Brain and muscle ARNT‐like 1
CLOCK: Circadian locomotor output cycles protein kaput
CRY: Cryptochrome
DCs: Dendritic cells
GF: Germ‐free
GM‐CSF: Granulocyte–macrophage colony‐stimulating factor
HIF: Hypoxia‐inducible factor
IECs: Intestinal epithelial cells
LPS: Lipopolysaccharides
MYD88: Myeloid differentiation primary response gene 88
NFIL3: Nuclear factor, Interleukin‐3 regulated
Nod2: Nucleotide oligomerization domain‐containing protein 2
PAMPs: Pathogen‐associated molecular patterns
PER: Period circadian protein homologue
PPARα: Peroxisome proliferator‐activated receptor alpha
PRRs: Pattern recognition receptors
ROR: Retinoic acid receptors
RORE: ROR response element
SCFA: Short‐chain fatty acids
SCN: Suprachiasmatic nucleus
SPF: Specific pathogen‐free
TLRs: Toll‐like receptors
TRIF: TIR‐domain‐containing adaptor‐inducing interferon‐β
ZT: Zeitgeber

## INTRODUCTION

**Figure 1:**
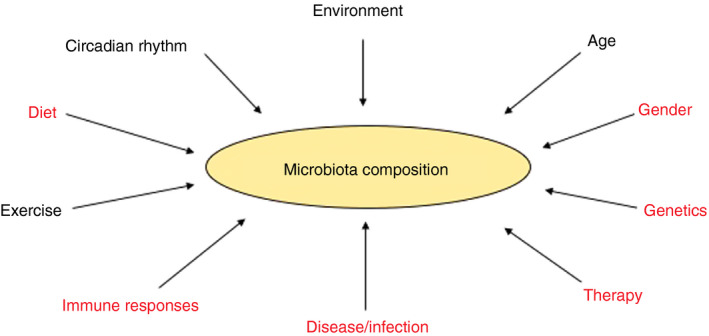
Factors influencing microbial composition. There are many factors which can alter microbial composition. Some factors can also modulate microbial composition and circadian rhythms, as discussed in this review.

The microbiota and the immune system have co‐evolved to maintain homeostasis and help protect our bodies from pathogens.^1^, ^2^ Approximately 1000 microbial species reside in the human intestines, encoding a metagenome of trillions of genes, which are over 100 times greater than the human genome, with millions of unique genes.^3^, ^4^, ^5^, ^6^ The microbiota influence many important aspects of host physiology, including modulating development, maturation and functions of the innate and adaptive immune systems. This has been highlighted in studies with gnotobiotic (mice with a defined microbial community) or germ‐free (GF; no microbiota) animals, which have a more naïve immune system, particularly in mucosa‐associated lymphoid tissues.^7^ The microbial composition can be modulated by a number of factors, including age, diet and host health (Figure 1). Disruption of the balance between the immune system and the microbiota promotes dysbiosis and increases susceptibility to various health issues including cancers, infections, autoimmune diseases and metabolic disorders.^8^, ^9^, ^10^, ^11^ Furthermore, microbiota can also modulate responses to immunotherapy, for example anti‐PD1 treatment in cancer.^12^, ^13^

**Figure 2:**
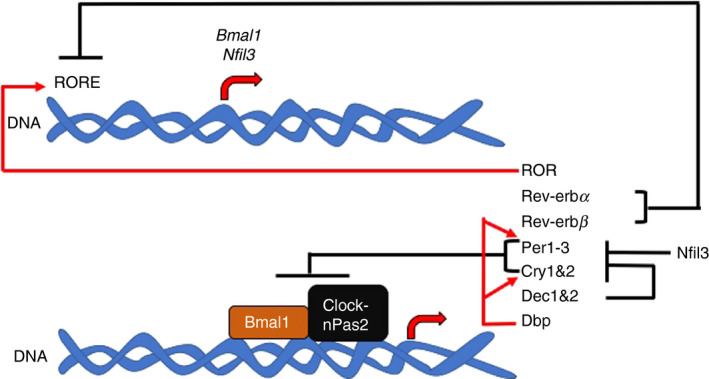
Circadian rhythm gene regulations. Initiation of the circadian rhythm requires activation of the circadian locomotor output cycles protein kaput (Clock), brain and muscle ARNT‐like 1 (Bmal1) and neuronal PAS domain‐containing protein 2 (nPas2) genes. These proteins regulate the expression of multiple genes – period circadian protein homologue 1, 2 and 3 (*Per1*‐*3*), cryptochrome 1 and 2 (*Cry1 and Cry2*), *Rev*‐*erbα* (*Nr1d1*), *Rev*‐*erbβ* (*Nr1d2*) and differentially expressed in chondrocytes protein 1 and 2 (*Dec1 and Dec2*). These proteins then repress the transcription of the *Clock*, *Bmal1* and *Npas2* genes, as well as their own transcription. Furthermore, D‐site of albumin promoter (Dbp) enhances transcription of clock genes, while nuclear factor, interleukin 3 regulated (Nfil3, also known as E4 bp4), suppresses clock gene transcription. Retinoic acid receptors (ROR) can promote *Bmal1* and *Nfil3* transcription from the ROR response elements (RORE). Red lines = gene activation; black lines = gene repression.

Circadian rhythms, referring to daily oscillations in gene activation and repression, as well as biological and physiological processes, can be induced by light, hormones, metabolic cues and the microbiota. The circadian rhythm influences immunity, microbial dynamics and host metabolism. Circadian rhythms are controlled by highly regulated transcription/translation feedback loops (Figure 2) and are reviewed in more detail elsewhere.^14^ Light is the main inducer of circadian rhythms via activation of the suprachiasmatic nucleus (SCN).^15^, ^16^ The SCN comprises ~20,000 specialized neurons within the hypothalamus, which control and co‐ordinate circadian rhythms in exercise, hormones, body temperature and eating. However, additional peripheral rhythms are needed for fine‐tuning the circadian clock, enhancing responses to environmental cues, for example food intake, body temperature and the microbiota. These peripheral rhythms differ from central circadian rhythms in that individual circadian clock components differentially modulate both types of rhythms. Furthermore, peripheral rhythms are subject to different influences which reset individual rhythms and control their outputs. Genes controlling these peripheral rhythms in different tissues also control individual cellular physiology.^17^, ^18^ Thus, peripheral rhythms temporally control many aspects of metabolism, including glucose homeostasis, lipogenesis and xenobiotic detoxification.^19^, ^20^, ^21^, ^22^, ^23^ These evolutionarily conserved, cell‐autonomous, biological clocks enable organisms to both anticipate, and adapt to, important environmental changes, aiding in their survival. In circadian studies, zeitgeber (ZT) measurements identify the time from the start of the rhythm to the end of the daily oscillation. In most cases, ZT times coincide with the number of hours after light exposure. For example, ZT12 refers to 12 hours after light exposure.

**Figure 3:**
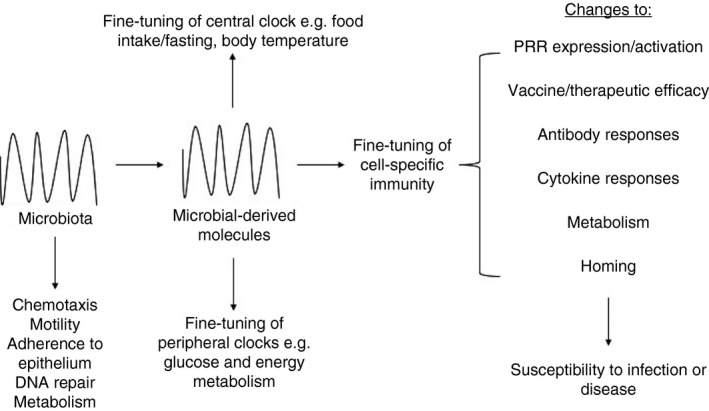
Importance of microbial oscillations on microbial functions. Microbial oscillations provide an important feedback system to both central and peripheral clocks through the oscillations in microbial metabolites, which help to fine‐tune the oscillations and ensure the host can respond more quickly to environmental changes. The oscillations in microbiota are also important for modulating their own functions including chemotaxis, motility, adherence, DNA repair and metabolism. The oscillations in microbiota and their metabolites are also vital for the fine‐tuning of immune responses. Oscillations in microbial ligands recognized by pattern recognition receptors (PRRs) are likely to result in differences in the strength of stimulation. Changes in microbial oscillations also strongly influence vaccine and therapeutic efficacy through modulating the immune functions at different times of day including antibody responses, cytokine responses, metabolic responses and homing of immune cells. Synchronization of therapy to the microbial rhythms is likely to provide the greatest efficacy, limiting susceptibility to infections and disease development.

In the intestine, circadian rhythms regulate digestion, including gastric acid production, gut motility and nutrient absorption. Circadian rhythms also affect intestinal stem cell regeneration and mucosal immunity.^24^, ^25^, ^26^, ^27^, ^28^ Moreover, the microbiota composition and functions depend on the time of day (Figure 3), giving rise to changes in susceptibility to disease. In this review, we discuss the changes in microbial composition and functions associated with circadian rhythms. We also discuss the mechanistic interactions between the microbiota and the immune system, and how they cross‐modulate daily oscillations. We highlight various associations that require further investigation, which will greatly enhance our knowledge and understanding of the cross‐regulation between microbiota, the immune system and circadian rhythms.

## MICROBIAL RHYTHMS ARE INFLUENCED BY NUTRIENTS AND THEIR AVAILABILITY

Similar to immune cells (discussed in the other review articles within this commissioned review series), the microbiota exhibit circadian rhythms, which modulate many important functions in the host (Figure 3). The microbial circadian dynamic is strongly associated with food intake and thus nutrient availability. In response to dietary glycans, Firmicutes thrive; however, once these glycans have been metabolized, Firmicutes decline in abundance, allowing Bacteroidetes and Verrucomicrobia to expand in response to accessible host glycans.^29^, ^30^, ^31^ These microbial rhythms promote metabolic homeostasis and can be modulated by food availability, which influences susceptibility to metabolic diseases, as discussed next.

### Time of food consumption drives microbial rhythmicity

In most laboratory animal facilities, mice are housed in 12‐hour light/dark cycles with food *ad libitum*. In these conditions, mice consume most of their food in the dark cycle, when they are most active.^32^ The composition of the caecal microbiota in mice, maintained under these conditions, changes dynamically in a cyclical manner, as shown by the microbial 16S rRNA sequence.^33^ Zarrinpar and colleagues found that the relative abundance of species belonging to the phylum Firmicutes peaked during the feeding period and was reduced during fasting, while species belonging to the phyla Bacteroidetes and Verrucomicrobia showed opposite trends (peaking during fasting and reducing when feeding).^33^ Similar changes have been reported by others.^30^, ^31^ Variations in microbial abundance are also associated with alterations in microbial functions. In the light phase, microbial pathways associated with chemotaxis and motility, which are important for microbiota to adhere to the intestinal wall, are increased.^30^, ^34^ In contrast, in the dark phase, pathways related to growth, DNA repair and energy metabolism are increased, which benefit the host by the production of growth factors including vitamins. These studies in mice indicate that microbially produced molecules can modulate immunity, as discussed later. Food restriction studies in mice, limiting food availability to either the light or dark cycle, have demonstrated that these microbial oscillations are similarly induced by food intake, regardless of whether in the light or dark cycle.^30^ Therefore, food availability and time of consumption drive microbial rhythmicity.

### High‐fat diet

Diet can modulate gut microbiota composition in humans.^35^, ^36^, ^37^ One of the most studied diets is a high‐fat diet, which alters the gut microbiota composition, promoting obesity and metabolic dysfunction in mice and humans.^38^, ^39^, ^40^, ^41^, ^42^ In mice, a high‐fat diet induces blunted microbial diurnal rhythmicity, compared with standard chow‐fed mice.^33^, ^42^ Interestingly, time‐restricted feeding of mice on high‐fat diet restored cyclical microbial rhythmicity and was able to protect from diet‐induced obesity and metabolic disease.^32^, ^33^ Importantly, in humans with metabolic disease or obesity, restricting food intake to 8‐12 hours per day resulted in weight loss and improved metabolic parameters.^43^, ^44^, ^45^, ^46^ It is unknown whether, as observed in the mice, the gut microbiota in these individuals was similarly altered; however, a recent study showed that the microbiota in individuals with obesity and type 2 diabetes had altered rhythmicity.^47^ Subsequent metagenomic analysis identified key altered microbial pathways, which associated with the clinical metabolic features of type 2 diabetes (e.g. fasting blood glucose, insulin resistance, HbA1c). Thus, the altered microbial functions could be used to predict the risk of disease development. Together, these studies highlight the importance of microbial circadian rhythms, and how they modulate disease in humans.

## HOST FACTORS ALTER MICROBIAL RHYTHMICITY

Microbial rhythmicity is strongly influenced by nutrient availability. However, when nutrients were administered only intravenously to mice (no oral intake), microbial rhythmicity was still observed,^42^ suggesting that host metabolic factors/rhythms, in turn, can also impact microbial rhythmicity. Many host functions are modulated by the circadian rhythm, including the secretion of hormones such as glucocorticoids and neurotransmitters, as well as immune regulation.^48^, ^49^ Importantly, immune cells express cell‐autonomous circadian rhythms.^50^, ^51^, ^52^, ^53^ Circadian rhythms alter immune cell differentiation, such as Th17 cell differentiation, which is regulated by Rev‐erbα‐driven repression of the *Rorγt* promoter via *Nfil3*.^54^ Homing receptors for immune cells, including CCR7, sphingosine‐1‐phosphate receptor 1, IL7‐R and CXCR4, are also modulated by circadian rhythms driven by the host glucocorticoids.^55^, ^56^, ^57^, ^58^ Thus, the circadian rhythm influences both innate and adaptive immune cell migration at different times of day. Together, these circadian rhythm‐modulated immune influences protect the host from disease development. As mentioned earlier, microbiota oscillate and thus may impact immune functions. For example, Th17 cells can be induced by segmented filamentous bacteria (SFB),^59^ which are members of the daily oscillating Firmicutes phylum.^30^, ^31^, ^33^ Furthermore, microbiota can modulate intestinal epithelial cell (IEC)‐intrinsic *Nfil3* circadian rhythms via type 3 innate lymphoid cells (ILC3).^23^ These observations suggest that microbial oscillations not only influence immune development, but also alter the amplitude of the type of immune responses. Thus, further understanding of circadian influences on host:microbial interactions is important.

### Host circadian factors modulate microbial rhythmicity

Mice deficient in key circadian rhythm genes, including *Bmal1* or *Per1*/*2*, have disrupted microbial rhythmicity and composition.^30^, ^31^ In addition, *Clock* gene mutant mice, which encode a dominant negative allele (*Δ19*) that alters the period, precision and persistence of circadian rhythms, also exhibit changes in microbial richness and diversity in the stool microbiota.^60^ These studies have demonstrated that the host circadian machinery influences microbial oscillations and composition; however, reciprocally, microbiota modulate host circadian rhythms. A comparison between GF (no microbiota present) and specific pathogen‐free (SPF; microbiota present but free of specific pathogens) mice confirmed that microbiota induce significant diurnal host circadian rhythms in liver hepatocytes.^42^ Interestingly, time‐restricted feeding can restore cellular circadian rhythms in *Cry1*/*2*‐deficient and liver‐specific *Bmal1* and *Rev*‐*erbα*/*β*‐deficient mice, preventing the development of metabolic syndrome and obesity.^61^ While microbial rhythms were not investigated in this study, it is likely that they were also altered. Thus, the crosstalk between microbiota and the host clock is important and together influences a number of systemic effects in the host, including fine‐tuning peripheral circadian rhythms.

### Androgens modulate microbial composition and rhythmicity

Some diseases have sex biases,^62^ suggesting androgens modulate disease development. In both mice and humans, androgens modulate the gut microbiota composition.^31^, ^63^, ^64^, ^65^ Interestingly, mouse studies showed that the gut microbiota modulate androgen metabolism,^66^ suggesting two‐way crosstalk. Androgens also modulate microbial oscillations. Liang and colleagues^31^ found more significant diurnal oscillations in female mice compared with the male mice. Furthermore, there were sex‐dependent differences in microbiota composition in *Bmal1*‐deficient mice.^31^ Sex differences also influence the circadian period and behaviour, including entrainment to light and food.^67^ Importantly, sex modulates circadian influences on many host physiological functions including blood pressure, body temperature and adrenal functions.^68^, ^69^, ^70^ Thus, it is imperative that any general conclusions, related to circadian influences, should be drawn from studies of both sexes in animals and humans, especially if a sex bias exists for the disease.

## CIRCADIAN MODULATION OF HOST PATTERN RECOGNITION RECEPTORS

**Figure 4:**
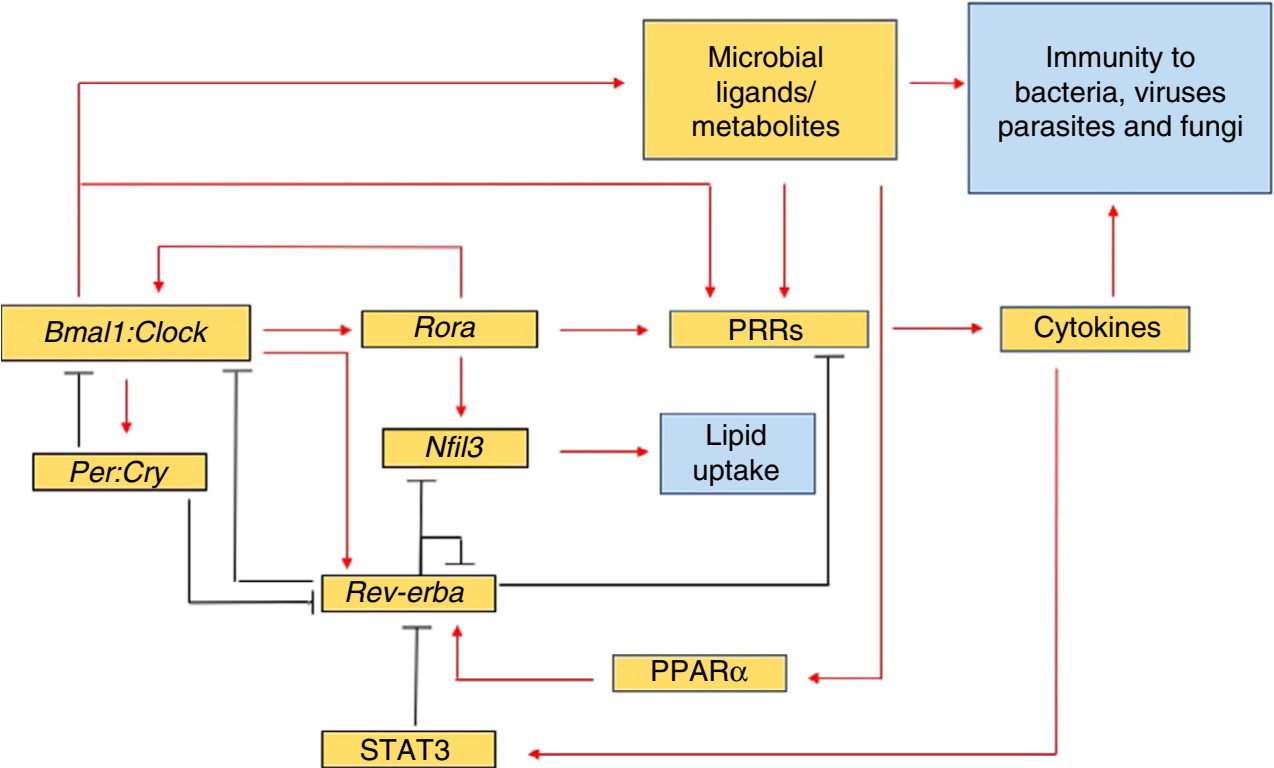
Microbial modulation of circadian rhythms. PRR oscillation (referring to published work in TLRs and Nlrp3) is driven by competition between the *Rev*‐*erbα* repressor and the *Rorα* activator, both of which directly bind and compete for the ROR response element, a DNA binding sequence, present in *Bmal1*. This competition promotes the oscillation of antimicrobial responses, for example cytokines, which are mediated by downstream NFκB and AP‐1 binding to target genes. Bmal1 binds to an Ebox element (a DNA sequence) in *Rev*‐*erbα* activating its transcription, while a DR2 element (another DNA sequence) in *Rev*‐*erbα* mediates its autorepression and activation by PPARα. DNA elements (RORE, Ebox, Dbox – not shown) are present in TLR genes, *Nod2*, circadian genes and many other genes, including many which influence immune processes. Circadian regulation of *Nfil3* by the *Rev*‐*erbα* repressor and the *Rorα* activator can also modulate intestinal lipid absorption and transport^23^. Another mechanism of circadian regulation of TLRs has also been identified in peritoneal macrophages, whereby *Tlr9* expression is controlled by direct binding of Bmal1 to the Ebox sequence present in the *Tlr9* gene^80^. Red lines indicate activation; black lines indicate inhibition. Blue boxes indicate the known effects of microbiota in modulating circadian rhythms.

Pattern recognition receptors (PRRs) are expressed on a wide variety of cells. These recognize conserved molecular structures that are shared by both pathogens and commensal microbes alike, and are known as pathogen‐associated molecular patterns (PAMPs).^71^ Through PRR signalling, the immune system is able to respond to microbial cues to regulate immunity. However, as discussed below, PRRs are also influenced by circadian rhythms (Figure 4), which can have important therapeutic effects, for example in the induction of vaccine‐induced immunity, as discussed later.

### Circadian modulation of Toll‐like receptor expression

Toll‐like receptors (TLRs) are the most commonly studied family of PRRs, found both at the cell surface and within intracellular endosomes. Each TLR recognizes different PAMPs, including bacterial/viral DNA (unmethylated cytosine‐phosphate‐guanine (CpG) dinucleotides^72^), lipopolysaccharides (LPS^73^) and viral RNA.^74^ While innate immune receptors prevent pathogen infection, they also have profound beneficial and detrimental impacts on the adaptive immune system, in both health and disease. This dichotomy is context‐dependent and has been observed in cancer, autoimmune and metabolic diseases, as reviewed elsewhere.^11^, ^75^, ^76^, ^77^, ^78^ While the effects of TLR signalling can be variable, depending on the disease and model studied, it is clear that TLRs are important immune targets for therapy.

TLR expression and activation occur in response to microbial cues and can be modulated by circadian rhythms, resulting in important functional differences in responses to microbes, at different times of day. TLR expression oscillates in macrophages, dendritic cells, B cells, T cells and non‐hematopoetic cells such as IECs.^23^, ^28^, ^50^, ^79^, ^80^ In macrophages, Silver and colleagues showed that different TLRs oscillate and peak at varying times; for example, the expression of *Tlr2* and *Tlr6* peaked at ZT19, while *Tlr4* peaked earlier at ZT15.^50^ As TLR2 can dimerize with TLR6,^81^ it is not surprising that these are co‐regulated to peak at the same time, whereas TLR4 is a homodimer and independently regulated and peaks at a different time. TLR4, however, binds to CD14,^82^ a co‐receptor. It is currently unclear whether coreceptors of PRRs, like CD14, are also regulated in a circadian manner. Furthermore, to our knowledge, there have been no studies correlating the rhythms of the microbiota with the abundance of microbial products that could activate PRRs. However, disruption to the gut microbiota by antibiotic treatment can dysregulate microbial rhythms, altering host circadian rhythms and TLR expression.^23^, ^28^, ^34^

Mukherji and colleagues found that the depletion of gut microbiota in mice resulted in reduced microbial recognition by TLRs, which dysregulated the IEC circadian clock.^28^ This increased ileal corticosterone production, altered glucose homeostasis and induced the development of prediabetes. Subsequently, Wang and colleagues found that microbial regulation of the IEC circadian clock was not directly mediated through the IEC.^23^ Previous studies had identified a subepithelial intestinal signalling relay, whereby bacterially mediated TLR activation of DCs induced the secretion of IL‐23, which in turn activated ILC3 cells to secrete IL‐22 and modulate IEC gene expression.^83^, ^84^ Wang and colleagues further confirmed that this DC‐ILC3‐IEC circuit was required for modulating IEC circadian rhythms (*Rev*‐*erbα* and *Nfil3*) and that the microbiota influenced the amplitude of the circadian rhythms in IECs through STAT3.^23^ Together, these data suggested that symbiosis between the microbiota and IEC requires intact circadian oscillations and a complex interplay between many cell types, which we are only beginning to decipher.

### Circadian modulation of other PRRs

In addition to TLRs, other PRRs, including the nucleotide oligomerization domain‐containing protein 2 (Nod2)^28^, ^85^ and the nod‐like receptor pyrin domain‐containing 3 (Nlrp3) protein, a component of the inflammasome, also oscillate. Both Nod2 and Nlrp3 can be activated by bacterial products, regulating the secretion of IL‐1β and IL‐18.^86^, ^87^, ^88^ Interestingly, in mice and humans, circadian oscillation of Nlrp3 is dependent on Rev‐erbα.^89^, ^90^ In mice, Rev‐erbα repressed *Nlrp3* transcription by specifically binding to the promoter region.^90^ Inflammasome components modulate responses to infections, as well as alter susceptibility to autoimmunity, neurological and metabolic diseases and cancer.^91^, ^92^, ^93^, ^94^, ^95^ Thus, further investigating the mechanisms of circadian modulation of PRRs may prove beneficial for therapy in a broad range of diseases.

## MICROBE‐DERIVED METABOLITES AND CIRCADIAN RHYTHMS

As microbiota composition and functions oscillate in a circadian manner, metabolites from gut microbiota^34^ in both mice and humans also exhibit circadian variations.^96^, ^97^, ^98^, ^99^, ^100^ Thaiss and co‐authors showed that antibiotic depletion of the microbiota led to the loss of microbial diurnal rhythmicity in mice.^34^ In addition, they also found that the microbiota‐induced oscillations in serum ornithine and polyamines could be induced by time‐restricted feeding, in a host that had deficient circadian rhythm (*Per1* and *Per2* knockout mouse). Together, these data provided pivotal evidence that microbiota modulate the host circadian metabolome. While this study did not investigate the impact of microbiota and their metabolites on host immunity, there are many potential implications for the immune system, especially mucosal immunity.

The gut microbe‐derived short‐chain fatty acids (SCFAs), butyrate, acetate and propionate, modulate immune responses, including the induction of Tregs.^101^, ^102^, ^103^ Interestingly butyrate, which is absent in germ‐free mice, modulates *Per2* and *Bmal1* rhythms, suggesting that the microbiota indirectly regulate circadian rhythms via their metabolites.^42^ These SCFAs typically bind to the G protein‐coupled receptors (Gpr) 41/43,^104^, ^105^ although other receptors also bind to SCFAs.^106^ Many types of host cells utilize SCFAs, and these promote deletion of autoreactive T cells and anti‐inflammatory responses in models of colitis, arthritis, type 1 diabetes and asthma.^104^, ^107^, ^108^ SCFA utilization can also restrict tumour growth and survival.^109^, ^110^, ^111^ Importantly, the effect of SCFAs on some cells, such as monocytes, may not always elicit the same responses from mice and humans.^112^ For example, activation of human monocytes with acetate induced attenuated proinflammatory responses; however, activation of mouse monocytes (on the 129/SvEv background) with acetate promoted elevated proinflammatory GM‐CSF, IL‐1α and IL‐1β cytokine secretion.^112^ Given that mouse and human monocytes respond differently to acetate, it is possible that additional regulators of SCFA utilization, and the rhythms that modulate them may vary between species. SCFAs such as butyrate can also regulate the integrity of the intestinal barrier by stabilizing hypoxia‐inducible factor (HIF).^113^, ^114^ Interestingly, HIFα regulates several core clock genes, allowing the cells to respond to changes in oxygen.^115^ This has implications, for example, for induction of tumour hypoxia in cancer therapy. Given that microbial metabolites oscillate, there may be similar oscillations in their respective SCFA receptors, or in proteins regulating the downstream responses.

Bile metabolism is also regulated in a time‐of‐day‐dependent manner due to the need to co‐ordinate metabolic responses to food intake, enabling the esterification and absorption of dietary fats and lipids.^116^, ^117^ In mice, the serum bile acids peak at the beginning and end of their active (dark) phase. The majority of bile acids are reabsorbed by the liver; however, some can enter the colon and be further metabolized, producing secondary metabolites that peak in the serum at the beginning of the dark phase.^118^, ^119^ Many species belonging to the phyla Firmicutes and Bacteroidetes encode enzymes required to metabolize bile acids, for example bile salt hydrolases. These enzymes regulate hepatic and ileal clock genes and lipid and cholesterol metabolism.^116^, ^117^, ^120^ Interestingly, the intestinal epithelium receptor, CD300lf, has been identified to be the receptor for mouse norovirus.^121^ This CD300lf receptor undergoes structural changes upon binding to the bile acid, glycochenodeoxycholic acid, which consequently enhanced the ability of norovirus to bind to the receptor.^122^ Recent studies have shown that microbiota can modulate the ability of mouse norovirus, a highly contagious viral pathogen, to infect the host.^123^ Furthermore, crosstalk between mouse norovirus and the microbiota can alter susceptibility to autoimmunity,^124^, ^125^, ^126^, parasitic infection^127^ and allergies.^128^ As bile acids can be modulated in a circadian manner, it is also possible that invasion of norovirus may be enhanced at different times of the day.

In addition, microbiota synthesize a range of other molecules including xenobiotics, vitamins, polyamines and hydrogen sulphide. Little is known about how circadian rhythms modulate their production and impact on the immune system. Investment in this area would aid our understanding of their important role in health and disease.

## IMPACT OF CIRCADIAN MICROBIAL OSCILLATIONS ON IMMUNITY TO INFECTIOUS ORGANISMS

Circadian rhythms, and their regulation, have an important influence on susceptibility to invading pathogens. Bellet and colleagues showed that the immune responses to *Salmonella Typhimurium* (a common foodborne pathogen) in infected mice were dependent on the time of day.^129^ The mice infected in the active phase (ZT16) had reduced pathogen colonization and reduced inflammation, compared with mice infected in the rest phase (ZT4).^129^ Host circadian rhythms are required for this response, as *Clock* mutant mice exhibited arrhythmic colonization and their macrophages had reduced inflammatory responses (IL‐6 and IL‐1β) to LPS (recognized by TLR4, which is known to oscillate^23^, ^28^, ^50^, ^79^, ^80^). Similarly, in wild‐type mice, *S*.*pneumoniae*‐induced infection at ZT12 resulted in earlier neutrophilia in the bronchoalveolar lavage fluid, with reduced local (lung) and systemic (blood) bacterial counts at 48 hours post‐infection, compared with infection at ZT0.^130^ In addition, intraperitoneal infection with *Listeria monocytogenes* confirmed that a later rest phase infection at ZT8 was important for enhanced recruitment of Ly6C^hi^ monocytes and stronger antimicrobial immunity, compared with infection at the start of the rest phase ZT0.^53^ Similar time‐of‐day‐dependent sensitivities to viral infections have also been reported.^131^, ^132^, ^133^ Interestingly, parasitic infection of mice by oral gavage of *Trichuris muris* eggs at ZT0 led to enhanced anti‐parasitic IgE titres, Th2 responses and worm expulsion, compared with the mice infected at ZT12, the end of the rest phase.^134^ Thus, the optimal immune responsive time to pathogens is not the same for all micro‐organisms. The different time‐dependent oscillations in pattern recognition receptors, or microbial metabolites, influence immune responsiveness. Therefore, priming the immune system at the time of greatest immune responsiveness would be vital for preventing and limiting the spread of specific infectious organisms. To date, it remains unknown as to whether the immune responses to these infectious organisms can be modulated by the commensal microbiota and whether the commensal microbial circadian rhythms are altered in pathogenic infections, which in turn subsequently change the host response. It is clear that further studies are required, especially when multiple factors are analysed in the same study.

## APPLICATIONS OF KNOWLEDGE OF MICROBIAL CIRCADIAN RHYTHMS TO THERAPY

The microbiome is an important metabolic ‘organ’, aiding in many unique host functions, which include the efficacious response to a number of therapies. It is well established that the microbiome influences susceptibility to obesity. Microbiota from obese individuals can transfer metabolic dysfunction to another host, while the microbiota from lean individuals, following transfer, protect the recipient from metabolic dysbiosis.^38^, ^135^, ^136^ Metformin, a widely used drug for the treatment of type 2 diabetes, improves insulin sensitivity. Metformin alters both the composition and the function of microbiota, enhancing the therapeutic effects.^137^ However, components of the gut microbiota can also be harmful, depending on the context. In colorectal cancer, the intestinal microbiota can influence disease progression^138^, ^139^, ^140^ or aid the therapeutic responses to both chemotherapeutics^141^, ^142^ and immune checkpoint inhibitors, for example CTLA‐4, PD‐1, PD‐L1.^13^, ^143^, ^144^ Therefore, understanding the mechanisms by which specific microbiota exert their effects (e.g. circadian modulation) could improve therapeutic success. In an animal model of colorectal cancer, induced by azoxymethane and dextran sulphate sodium, Mager and colleagues identified 3 species of tumour‐associated bacteria (*Bifidobacterium pseudolongum*, *Lactobacillus johnsonii* and *Olsenella sp*.) which enhanced immune checkpoint blockade therapy.^145^ This enhancement of therapy was orchestrated predominantly by the microbial production of inosine, which binds to the adenosine A_2A_ receptor, promoting Th1 differentiation in the presence of IFN‐γ and consequently anti‐tumour effects. Of note, *Lactobacillus johnsonii* belongs to the phylum Firmicutes, the abundance of which is known to oscillate daily.^30^, ^31^, ^33^ *Lactobacillus johnsonii* is also associated with modulating immunity and preventing autoimmune diabetes.^146^, ^147^, ^148^ In a recent study assessing metabolism of 271 orally administered drugs by 76 different human gut bacteria, the authors reported that the microbiota can metabolize many more drugs than previously known.^149^ In addition, the drug‐metabolizing function of the microbiota has both local intestinal effects and important systemic effects, especially on the liver. Hepatic drug metabolism is also influenced by circadian rhythm.^150^, ^151^, ^152^ Thus, circadian microbial oscillations (whether through direct or indirect mechanisms) are likely to modulate the ability to metabolize drugs and therefore may have important therapeutic impacts. It should also be noted that peripheral circadian rhythms are often tissue‐specific, and therefore, microbial rhythms could be out of synchrony with the rhythms in different tissues.^17^, ^18^ This may potentially arise due to the presence of dysbiotic microbiota or stronger peripheral circadian inducers. Thus, it is critically important to identify novel pathways regulating the peripheral rhythms, which are influenced by the microbiota, and synchronize these rhythms, for maximal clinical benefits.

The types of gut microbiota that adhere to the intestinal wall may also oscillate and can play an important role in intestinal immunity. *Akkermansia muciniphilia*, a mucin‐degrading commensal bacteria belonging to the Verrucomicrobia phylum,^153^ is protective against ulcerative colitis,^154^, ^155^, ^156^ type 1 diabetes^157^, ^158^, ^159^ and obesity.^160^ Furthermore, *Akkermansia muciniphilia* improves therapeutic efficacy in cancer patients who are treated with immune checkpoint blockers.^13^ Given the oscillation in abundance of Verrucomicrobia,^30^, ^31^, ^33^ appropriately timing therapeutic administration of these bacteria may enhance efficacy. As discussed earlier, it is important to note that bacteria can be beneficial or harmful, depending on the context. *Akkermansia muciniphilia*, while beneficial in the aforementioned studies, can also promote T‐cell‐mediated inflammation in multiple sclerosis^161^ and thus microbial therapies need to be tailored appropriately.

Microbial circadian rhythms modulate long‐term immunity following immunization and vaccination. Microbiota composition can modulate vaccine efficacy to bacteria and viruses in both animal models^162^, ^163^, ^164^ and humans.^165^, ^166^, ^167^, ^168^ Evidence for this has been obtained from vaccine studies conducted in GF, as well as antibiotic‐treated mice, resulting in impaired antibody responses.^162^, ^163^ Similarly, in humans, administration of broad‐spectrum antibiotics also reduced the immunogenicity of the rotavirus vaccine.^167^ Rhythmic microbial and PRR rhythms are also likely to influence vaccine efficacy, although this remains to be studied. Host circadian genes can also alter the vaccine efficacy. *Per2*, one of the major circadian gene family members,^169^ controls TLR9‐mediated innate and adaptive immune responses to infection and sepsis.^80^ Moreover, *Per2* also controls vaccine immune responses to TLR9 ligand‐adjuvanted immunization using CpG (a bacterial DNA, which is a TLR9 ligand and a common vaccine adjuvant^72^).^80^ These studies have provided evidence for a vital link between circadian rhythms and TLR signalling, which promotes enhanced vaccine efficacy. Furthermore, in *Cry1*/*Cry2* double knockout mice, there are more circulating mature B cells that secrete higher titres of antibodies to T‐cell‐independent antigenic stimulation (4‐hydroxy‐3‐nitrophenyl‐acetyl (NP)‐conjugated Ficoll).^170^ This again highlights host rhythms influencing immunity. Oscillations in antibody responses have also been seen in other studies, including vaccination studies.^56^, ^171^, ^172^, ^173^, ^174^ A randomized trial investigating the efficacy of the trivalent inactivated influenza virus demonstrated that vaccine administration in the morning induced higher antibody titres than in the afternoon^173^; however, the oscillating antibodies post‐vaccination are likely to be viral strain‐specific^171^ and influenced by sex.^172^ As TLR9 is highly expressed on B cells^175^ and requires downstream MyD88 signalling to mediate functional changes,^176^, ^177^ it is likely that oscillating TLR9 levels may influence B‐cell antibody responses; however, this has yet to be fully investigated. This also applies to autoantibody production, which can be regulated by TLR expression.^178^, ^179^, ^180^ Murine studies, in which peptide immunization was performed at different times of day, showed that this influenced both T‐cell immunity and susceptibility to disease.^56^, ^181^, ^182^ Furthermore, immune‐intrinsic circadian rhythms were vital for this effect.^56^, ^182^ Thus, both Bcell and Tcell responses to antigen can be modulated by circadian rhythms. Further understanding of how the microbial rhythms may influence antigen‐specific immunity may prove even more beneficial.

While the role of microbiota‐mediated modulation of host immunity using antibiotic treatment or GF mice has been extensively studied, the impact of pro‐ or pre‐biotics or bacteriophages (bacteria‐targeting viruses) on either host or microbial circadian rhythms is not currently known. Studying bacteriophages and their role in modulating microbial circadian rhythms may therefore also prove to be an important research field, particularly as bacteriophages can adhere to the mucus layer and prevent infection from pathogenic bacteria.^183^, ^184^

## CHALLENGES IN STUDYING MICROBIAL RHYTHMICITY

### Reporting microbial rhythmicity

A common challenge facing researchers studying the microbial rhythmicity lies in the interpretation and presentation of the data. Relative microbial abundance is often reported in microbial circadian oscillation studies, referring to the proportion of specific bacteria within the total bacteria sequenced. However, this can potentially under‐ or over‐estimate changes in the microbiota composition and thus the inferred absolute microbial abundance is sometimes used. The inferred absolute microbial abundance is determined by multiplying the 16S rRNA copy number by the relative abundance within a given sample. Studies using different methods could lead to discrepancies between different reports of the abundance of bacteria which oscillate at different times of day. For clarity, we suggest that the reporting of these microbial rhythms should be presented both ways. An alternative method would be to conduct both microbial sequencing and a culture‐based approach (on selective media) to identify numbers of specific bacteria; however, this approach is not infallible as most gut bacteria cannot be cultured. Further developments are needed in this area before true microbial counts for all bacteria can be determined.

### Limited studies in both sexes

As previously mentioned, sex influences the microbial composition and rhythmicity. While some studies do investigate sex biases, many more do not. Thus, studies should be conducted in both males and females to identify the role of androgens/oestrogens in modulating circadian rhythms.

### Evaluating all variables

There are many factors, already discussed, that can influence microbial composition and circadian rhythms, including light, diet, therapies (e.g. antibiotics), genetics, hormones, sleep patterns, behaviour and disease development. In many studies, it is difficult to investigate all variables; however, in mice, many of these can be controlled but not all are studied. This is the same in human studies, where food intake is restricted^44^, ^45^, ^46^ but exercise, genetics, light exposure, among other factors, are not considered. While these studies highlight the importance of circadian rhythms in health and disease, future studies need to consider additional factors and their contribution, if any, to the results obtained. Larger study cohorts and controlled facilities, where all participants reside and are maintained in the same light cycles and on the same diet, are likely to be required.

## SUMMARY

In this review, we have discussed time‐of‐day‐dependent differences in the microbiota and how they may impact host metabolism and immunity. While it is known the microbiota are associated with the development and progression of many diseases, little is known of the influence that circadian oscillations have on the microbiota, which may also modulate disease susceptibility. We hope to have provided insight into some potential future directions in relation to microbial oscillations and how they can directly, or indirectly, regulate host immune responses in different disease settings. Given the significant influence that circadian rhythms have on host immunity, it is important that more studies consider the timing of experiments and administration of therapies. By better understanding circadian influences, we may maximize clinical success by targeting the cells/microbiota of interest, at the time they are most vulnerable. This could potentially enable reduced drug concentrations to be used, which would also limit toxic effects. Understanding these rhythms in both males and females is vital, given the importance of androgens in immunity and in shaping the host microbial communities. While the vast majority of work outlined in this review has focused on microbiota in the gut, it will also be important to study the circadian effects on microbiota in different locations that include the skin, lungs, as well as other mucosal surfaces. It is likely that most commensal microbiota, in these different locations, modulate immunity in a time‐of‐day‐dependent manner. This is a continually expanding new field and we look forward to gaining further insight, which may improve efficacy of current, as well as new therapies, for disease prevention and treatment.

## FUNDING INFORMATION

This work was supported by a Medical Research Council Career Development Award (MR/T010525/1) to JAP; a NIH Research Project Grant (NIH RO1 HD097808); and a Molecular Genetic & Diabetes Mouse Core of Yale Diabetes Center grant to LW (NIH P30 DK 045735).

## CONFLICTS OF INTEREST

The authors declare no conflicts of interest.

## Data Availability

Data sharing not applicable to this article as no datasets were generated or analysed during the current study.
